# The Dissection Bill

**Published:** 1854-05

**Authors:** 


*The Dissection Bill.*—As intimated elsewhere, this bill has become a law. We hoped to secure a copy for publication in our present issue, but are unable to do so this month. It has been very much amended and changed from its first form. Many of its provisions are vexatious and insufficient, but these may be corrected as they make themselves manifest. In the mean time we believe the friends of the bill in the legislature did all in their power to make it fair and liberal. Any restrictions of a contrary tendency were submitted to, not approved, by them, in order to secure the passage of the bill. For this they deserve the thanks of the profession, and though we could wish for a better law, we owe a debt of gratitude to those whose prudence has been equal to their zeal in our behalf. H.

